# Are there differences in abomasal morphology in calves fed eight liters of milk and concentrate or good quality alfalfa hay?

**DOI:** 10.29374/2527-2179.bjvm002825

**Published:** 2025-09-26

**Authors:** Noelia Vazquez, Dellis dos Santos, Germán Antúnez, Nicolás Amaro, Josefina Escardó, Lucía López, Cecilia Cajarville

**Affiliations:** 1 Unidad de Anatomía- Facultad de Veterinaria, UdelaR, Montevideo, Uruguay; 2 Departamento de Ciencias Veterinarias y Agrarias-CENUR-LN, Facultad de Veterinaria, Universidad de la República, EEMAC, Paysandú, Uruguay; 3 Departamento de Producción Animal y Salud de los Sistemas Productivos, Facultad de Veterinaria, Universidad de la República, Libertad, San José, Uruguay; 4 Unidad Académica de Genética y Mejora Animal, Departamento de Producción Animal y Salud de los Sistemas Productivos, Facultad de Veterinaria-Udelar, Montevideo, Uruguay; 5 Autonomous veterinarian, Montevideo, Uruguay

**Keywords:** accelerated breeding, ruminants, stomach, criação acelerada, ruminantes, estômago

## Abstract

This study investigated the anatomical and histological characteristics of the abomasum in weaned Holstein calves under two feeding regimens: high-quality alfalfa hay and starter concentrate. The calves were raised under an accelerated feeding system (milk diet provided at 20% of their body weight). This study aimed to determine the effects of diet on the development of the abomasum during the early life stages of calves, which is crucial for digesting both milk and solid feed. Twenty newborn male Holstein calves were randomly assigned to either diet group and monitored from 56 to 60 days of age. Organ weight, abomasal curvature length, and number of abomasal folds were measured. Histological slides were prepared for microscopic examination. No significant differences in anatomical and histological parameters were observed between the two dietary groups, suggesting that both diets provided adequate nutritional support for development. Although the quantity of feed differed, the primary component, milk, remained consistent, contributing to balanced nutrition. This study emphasized the importance of early nutrition in calves and its influence on the anatomical and histological development of the abomasum. However, potential subtle differences warrant further investigation to determine long-term consequences on digestive health and efficiency.

## Resumo

Este estudo investigou as características anatômicas e histológicas do abomaso em bezerros Holstein desmamados sob dois regimes alimentares: feno de alfafa de alta qualidade e concentrado inicial. Os bezerros foram criados sob um sistema de alimentação acelerada (dieta láctea fornecida a 20% do seu peso corporal). Este estudo teve como objetivo determinar os efeitos da dieta no desenvolvimento do abomaso durante os primeiros estágios de vida dos bezerros, que é crucial para a digestão do leite e da ração sólida. Vinte bezerros Holstein recém-nascidos foram aleatoriamente designados para um dos grupos de dieta e monitorados dos 56 aos 60 dias de idade. O peso do órgão, o comprimento da curvatura do abomaso e o número de pregas do abomaso foram medidos. Lâminas histológicas foram preparadas para exame microscópico. Não foram observadas diferenças significativas nos parâmetros anatômicos e histológicos entre os dois grupos alimentares, sugerindo que ambas as dietas forneceram suporte nutricional adequado para o desenvolvimento. Embora a quantidade de ração fosse diferente, o componente principal, o leite, permaneceu consistente, contribuindo para uma nutrição equilibrada. Este estudo enfatizou a importância da nutrição precoce em bezerros e sua influência no desenvolvimento anatômico e histológico do abomaso. No entanto, possíveis diferenças sutis justificam uma investigação mais aprofundada para determinar as consequências a longo prazo para a saúde e a eficiência digestiva.

## Introduction

A ruminant is born with a digestive tract resembling that of a nonruminant in terms of structure and function. At birth, the stomach of a calf is prepared for milk digestion. At this stage, digestion occurs in the abomasum and intestines and resembles the monogastric digestion process (Diao et al., 2019). Therefore, the digestive tract of calves and their passage to functional ruminants should develop during the lactation stage (Khan et al., 2016).

Calves can be raised naturally (at the mother's foot where they can suck *ad libitum*) or artificially (using traditional or accelerated feeding systems). In the traditional breeding system, calves are provided 8-10% of their body weight (BW) of milk or milk replacer (MR) daily (approximately 4 l) in two doses (Terré et al., 2007). A starter concentrate is added from the first days of life, in the order of 0.2% to 0.25% of BW until reaching a consumption of 1 kg of food daily for 3 days, where milking is performed. This period lasts for approximately 60 days (Lagger, 2010). These starter feeds are crucial for initiating rumen development and allowing early weaning; therefore, ensuring that they are palatable and well pelleted and contain high nutritional value is paramount (Quigley, 1997). In this type of breeding, the goal is using the minimum amount of dairy to reduce costs and avoid affecting the health of the calves.

By contrast, the accelerated feeding system is based on the calves’ natural behavior, applying the principles of animal welfare and supplying quantities similar to what the calves would naturally drink from their mothers. Generally, this represents nearly 20% of the calves’ BW, equivalent to twice more milk than that used in traditional feeding systems, and provides a concentrate (Raeth-Knight et al., 2009).

Independent of the feeding system used, many anatomical, histological, and microbial changes occur during this period to prepare young ruminants for the proper digestion of solid feed (Davis & Drackley, 1998; Khan et al., 2016).

Histologically, all stomach divisions have four typical layers of tubular organs in the digestive system. The abomasum contains mucus for protection owing to its low pH, especially when the organ is empty. The coiled glands present in the lamina propria open into the gastric pits. Rugae are present in the pyloric region, and a pyloric torus narrows the lumen (Ali et al., 2023).

Evidence regarding the histomorphological characteristics of the abomasum according to diet is scarce. Moreover, comparative anatomical studies of different ruminant species have revealed significant differences. The empty weight of the abomasum, expressed as a function of live weight and weight of the entire digestive system, is greater in sheep than in chamois *(Rupicapra rupicapra*), whereas ibex (*Capra ibex)* have intermediate values for these variables (Lavín & Mantecón, 1993).

Some histological differences have been observed in the development of the epithelium, number of glands, and keratinization level of the abomasum of lambs and goats fed diets with different concentrations of tannins (Kia et al., 2016; Mbatha et al., 2002). This supports the idea that diet can influence the anatomical characteristics of the abomasum.

In calves, the abomasum is critical for the digestion of ingested milk and participates in the digestion of solid foods fermented in the rumen. Most studies on dairy calves have focused on the characteristics of liquid diets (milk, MR, or rehydration salts) and the physiology or pathological processes of the abomasum (Burgstaller et al., 2017). Different factors of a liquid diet, such as volume, osmolality, viscosity, glucose content, or caloric content, affect the abomasal emptying rate in calves (Burgstaller et al., 2017). However, whether the type of solid food affects the histological and anatomical characteristics of suckling calves that have access to large volumes of milk or MRs remains unknown.

Therefore, this study aimed to evaluate the effect of high-quality alfalfa hay or starter concentrate fed as a solid feed in calves raised under an accelerated feeding system (milk diet provided at 20% of their BW) on the anatomical and histological characteristics of the abomasum at 60 days old of weaned dairy Holstein calves.

## Materials and methods

The experimental trial was conducted at the Instituto de Producción Animal de la Facultad de Veterinaria, Universidad de la República (UdelaR) located on route 1 km 42, Libertad, San José, Uruguay (34°40’40”S 56°32’13”W). Throughout the experimental period, the average air temperature was 22.1 °C, and the average relative humidity was 66.2% (data reported by de Instituto Uruguayo de Meteorología). Twenty newborn male Holstein calves with adequate passive transfer of immunity (serum refractometer reading > 8.4%; Deelen et al., 2014) were used. Calves were individually housed in pens (2 m × 1 m) under controlled sanitary and environmental conditions. The animals were blocked by initial BW and week of birth and then randomly assigned to one of two treatments: (1) 8 l of MR with *ad libitum* access to alfalfa hay or (2) 8 l of MR with *ad libitum* access to starter concentrate feed. All calves received MR diluted to 12% dry matter (DM) at 38 ± 1 °C, administered in two equal meals at 08:30 and 16:30 h. During week 8, calves in both treatment groups were gradually weaned, and MR was discontinued on day 57 of life. During weeks 9 and 10, the calves had *ad libitum* access to alfalfa hay and starter concentrate feed and remained in the same pen.

The feedstuffs (Table 1) were analyzed to determine the DM (method, 934.01), ash (method, 942.05), crude protein (CP; method, 955.04), and ether extract (method, 920.39; Association of Analytical Communities, 2000) contents. Neutral detergent fiber (NDF) was performed using thermostable α-amylase and sodium sulfite, whereas acid detergent fiber was performed non-sequentially (Van Soest et al., 1991). For both NDF and ADF, the residual ash content was discounted (Licitra et al., 1996; Van Soest et al., 1991). Metabolizable energy content was estimated according to the National Research Council (2001).

**Table 1:** Nutrient compositions of milk replacer (MR), concentrate (CON), and alfalfa hay (ALF).

**Variables**	**Milk replacer**	**Concentrated**	**Alfalfa hay (forage)**
DM (%)	95.3	90.0	90.5
Crude Protein (% DM)	21.5	18.0	16.0
NDF (% DM)	−	17.9	40.4
ADF (% DM)	−	7.2	32.4
Ash (% DM)	5.6	5.6	7.2
Ether extract (% DM)	20.0	3.4	-
ME (Mcal/kg DM)^1^	4.58	3.00	1.96
Lactose (% AF)^2^	44.0	-	-
Calcium (% AF)*	1.3	-	-
Total phosphorum (% AF)*	0.6	-	-
Sodium (% AF)*	0.4	-	-
Chlorine (% AF)*	0.5	-	-
Inorg. copper (ppm)*	11.0	-	-
Inorg. zinc (ppm)*	44.0	-	-
Iron (ppm)*	111.0	-	-
Vitamin A (IU/Kg AF)*	27000	-	-
Vitamin D3 (IU/Kg AF)*	5300	-	-
Vitamin E (IU/Kg AF)*	50000	-	-
Ionophore (ppm/Kg AF)*	100000	-	-
Total lysine (% DM)*	2.7	-	-
Total Methionine (% DM)*	0.9	-	-
Aflatoxins B1, B2, G1, G2 (ppb)*	-	< 5	-
DON (ppb)*	-	< 500	-
Zearalenone (ppb)*	-	< 50	-

The variables indicated with * correspond to values reported on the product label.

1 Metabolizable energy was calculated according to Drackley (2008) for milk replacer, and according to National Research Council (2001) for concentrate and alfalfa hay;

2 Lactose were estimated as: DM-(CP+EE+Ash).

DM (Dry Matter), NDF (Neutral Detergent Fiber), ADF (Acid Detergent Fiber), AF (As-Fed), ME (Metabolizable Energy), DON (Deoxynivalenol)

MR and solid feed intake (forage or starter feed) were recorded daily, and BW was measured weekly throughout the 10-week experiment. The proportion of forage in the diet was calculated as the proportion of the total concentrate and forage intake. BW and total weight gain over 10 weeks were adjusted for gut fill, as described by Antúnez-Tort et al. (2023). Gain-to-feed efficiency was calculated as the difference between final and initial BW, both corrected for gut fill, divided by the total feed intake over the 10-week study period and expressed as a percentage.

After maintaining the animals under the experimental conditions of the group to which they belonged, they were euthanized after 2 h of fasting. The animals were sacrificed using a captive bolt gun and subsequently exsanguinated through an incision of the jugular vein and carotid artery. The method was approved by the Comisión Nacional de Experimentación Animal (protocol no. 685) of the Facultad de Veterinaria, UdelaR.

### Study methods

To examine the abomasum, dissection was first performed. The length of the greater and lesser curvatures, height of the pyloric torus, weight of the empty and full organs, and number of abomasal folds were measured. To avoid bias, all measurements were performed by the same researcher.

The abomasum was first isolated from the other digestive organs to obtain the corresponding measurements. Double ligation was performed in the region close to the omaso-abomasal orifice and pyloric region. All omentals were removed. The organs and their contents were then weighed. Next, the organs were emptied, washed with running water, and allowed to drain for 10 min before weighing. Finally, the lengths of both the curvatures were measured using a meter. The organs were preserved in a container with 4% formalin to count the abomasal folds and measure the pyloric torus.

### Collection and processing of samples for scanning electron microscopy and optical microscopy

Samples were obtained from two animals in each group, 1 cm long and 1 cm wide, from each region of the abomasum (fundus, body, and pyloric region). The samples were carefully collected using toothless forceps and washed by immersing them in water at 5 °C. The samples were then placed in a 4% paraformaldehyde solution. The bottle in which the samples were stored contained 10 times the volume of the fixative relative to the size of the sample. The samples were stored at room temperature for at least 10 days.

For scanning electron microscopy, the samples were washed with 1% phosphate-buffered saline solution in three baths for 5 min and three baths with distilled water for 3 min. They were then dehydrated in serial concentrations of alcohol (50%, 70%, 90%, and 100%), dried in a critical-point apparatus, glued with carbon glue to aluminum metal bases, and metalized with gold in a metallizing apparatus. Finally, they were analyzed and photographed using a scanning electron microscope at the Facultad de Ciencias of UdelaR-Montevideo (Uruguay).

### Histology

Histological slides of the abomasum samples were prepared at the Laboratory of the Universidade Federal de Santa Maria, Brazil. Samples were collected from the fundus, body, pyloric region, pyloric torus, and abomasal folds. All samples were processed using routine paraffin embedding. Tissue samples were cut at 5-μm thickness and mounted on slides. Two slides of each sample were prepared. The slides were stained with hematoxylin and eosin. Images from each slide were captured at 4×, 10×, 20×, and 40× magnification.

### Statistical analysis

Data obtained were analyzed to confirm that they followed a normal distribution. Because N was small, the Shapiro–Wilk test was used. The t-test for two independent samples was used to compare the quantitative results of the two groups. The total of each feed ingested, final BW, and total kg of BW gained were analyzed using a generalized linear mixed model by GLIMMIX procedure in SAS (SAS Institute Inc., Cary, NC). The model included treatment as a fixed effect, block as a random effect, and initial BW as a covariate. Least squares mean among treatments were compared using the Tukey–Kramer adjustment, and the standard error of the mean is reported for each comparison. Statistical significance was set at P ≤ 0.05. The data obtained are expressed as means and standard deviations.

## Results

During the study, calves consumed a similar amount of MR. However, calves in the starter feed treatment group consumed more starter feed (P < 0.01), greater amounts of total solid feed and total DM (P < 0.01), and less alfalfa hay (P < 0.01). The final BW, BW gain over the 10-week period, and gain-to-feed efficiency did not differ between treatments (Table 2).

**Table 2:** Total feed ingestion and performance throughout the 10-week experiment.

**Item**	**Treatments** 1		**SEM**	***P*-value**2
	**Alfalfa**	**Starter**		
**Feed ingestion (wk 1-10 of life)**				
Total MR intake, kg DM	46.4	46.7	0.52	0.915
Total starter DM intake, kg	14.4	37.8	3.11	< 0.01
Total alfalfa hay DM intake, kg	17.0	5.7	1.56	< 0.01
Total solid feed DM, 3 kg DM	31.2	43.3	3.41	< 0.01
Total DM intake, 4 kg	108.8	133.2	7.10	< 0.01
Proportion of forage, 5 %	54.5	13.2	2.76	< 0.01
Forage/Concentrate, %	1.18	0.15		< 0.01
**Performance (wk 1-10 of life)**				
Final BW, 6 kg	77.0	80.5	3.09	0.246
BW gained, ^6^ kg	36.9	40.3	3.08	0.246
Gain-to-feed, ^7%^	34.8	31.1	2.65	0.229

1 Treatments: free access to alfalfa hay or starter feed as solid feed before weaning (wk 8), and free access to alfalfa hay and starter feed from week 9 to 10 of the study;^2^Effect of treatment (P ≤ 0.05);^3^Total solid feed intake: sum of alfalfa hay and starter feed intake;^4^As sum of DM intake from milk replacer, alfalfa hay, and starter feed;^5^Forage intake as a proportion of the sum of concentrate and forage consumed;^7^Adjusted for gut fill similarly to Antúnez-Tort et al. (2023).

SEM (Standard Error of the Mean), DM (Dry Matter), BW (Body Weight)

**Figure 1:**
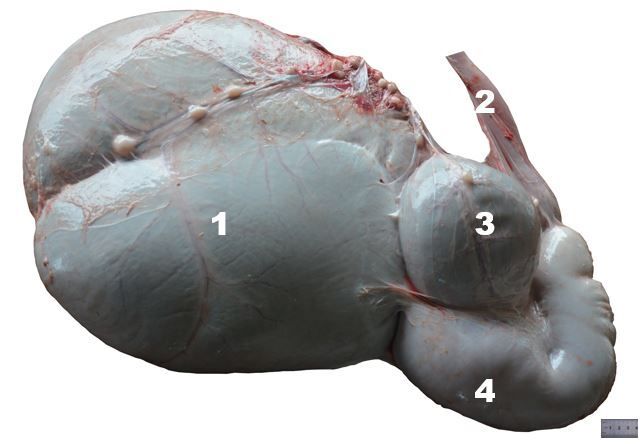
The stomach of a Holstein calf fed 8 l of milk replacer per day and forage *ad libitum*. (1) ventral sac of the rumen; (2) duodenum; (3) omasum; (4) abomasum.

**Figure 2:**
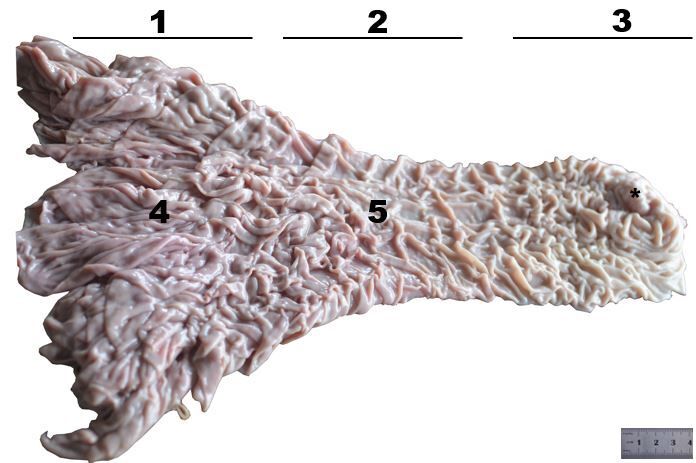
The abomasum of a Holstein calf fed 8 l of milk replacer per day and *ad libitum* forage. (1) fundus; (2) body; (3) pyloric region; (4) abomasal folds; (5) spiral folds. *pyloric torus.

The studied abomasums showed typical structures of this organ. Their arrangement and shape were also typical (Figure 1). The presence of glands in all portions of the abomasum, development of abomasal folds, and pyloric tori were observed in both groups (Figure 2).

**Figure 3:**
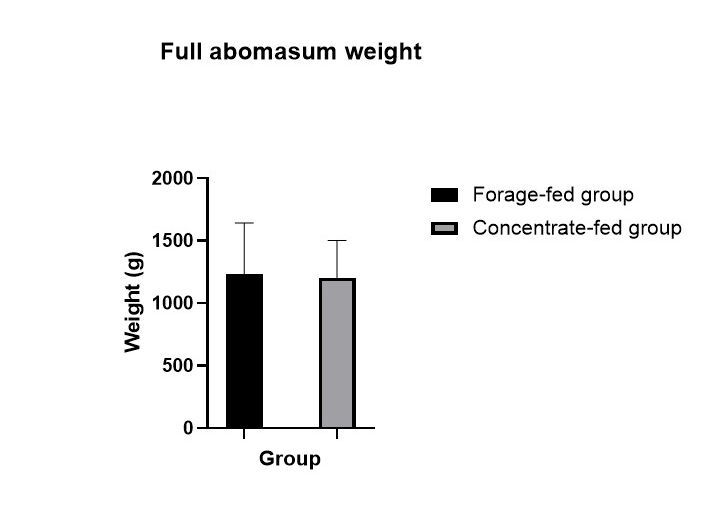
Graph comparing the weight of the full abomasum between the group of Holstein calves fed 8 l of milk replacer per day and forage or concentrate *ad libitum*.

**Figure 4:**
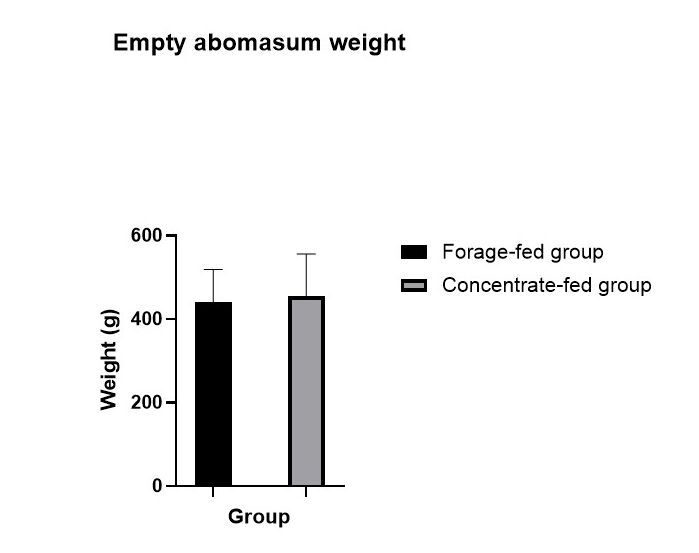
Graph comparing empty abomasal weight between the group of Holstein calves fed 8 l of milk replacer per day and *ad libitum* forage or concentrate.

The weight of the filled abomasum was 1206.6 g (±297.53 g) in the concentrate-fed group and 1232.5 g (±411.97 g) in the other group. The empty organ weight was 454.41 g (±101.8 g) in the first group and 439.84 g (± 79.02 g) in the other group (Figures 3 and 4). No significant differences were observed between these values.

The percentage of BW that represented the full abomasum was 1.31% in the concentrate-fed group and 0.50% in the empty group. Those of the hay-fed group were 1.4% full and 0.51% empty, respectively, with no significant differences.

**Figure 5:**
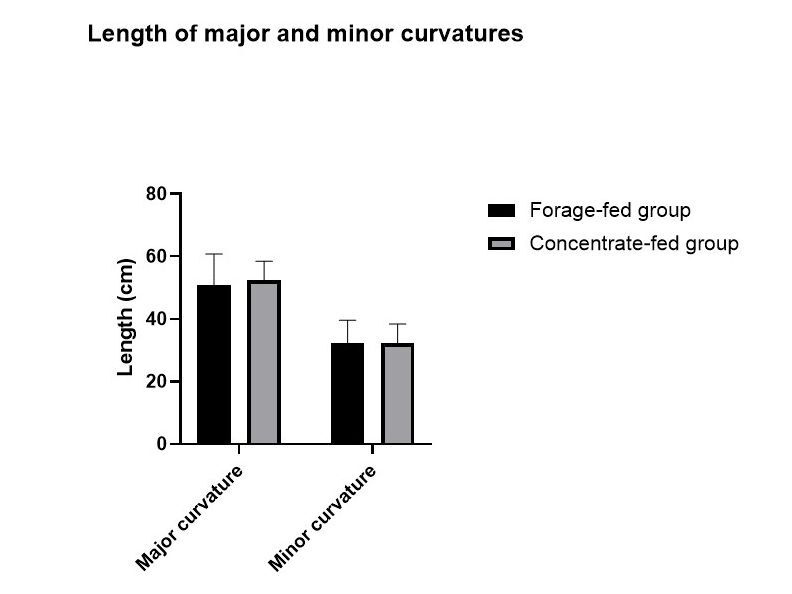
Graph comparing the length of the major and minor curvatures between the group of Holstein calves fed 8 l of milk replacer per day and *ad libitum* forage or concentrate.

The measurements of the greater (50.7 cm in the hay-fed group and 52.4 in the concentrate-fed group) and lesser (32.2 in the hay-fed group and 32.3 in the concentrate-fed group) curvatures of the abomasum did not show significant differences between the two groups of calves (Figure 5).

The parameters referring to the abomasal folds and height of the pyloric torus in both groups of animals are detailed in Table 3, along with their respective means, standard deviations, and T test. Pyloric torus did not develop in three animals (one from the hay-fed group and two from the concentrate-fed group).

**Table 3:** Height of the pyloric torus and number of abomasal folds of dairy calves that received concentrate (CON) or alfalfa hay (ALF) as a solid feed expressed as means (Ms) and standard deviations (SDs).

	**Value**	**M ALF**	**SD**	**M CON**	**SD**	**T-test**
Abomasal Folds	Number	15.3	4.06	16.4	3.98	The t-value is -0.61227. The *p-value* is 0.27400
Height of the pyloric torux	mm	14.89	4.2	13.25	3.24	The t-value is 0.89215. The *p-value* is 0.193202

M (Mean), T-test (Student’s t-test)

**Figure 6:**
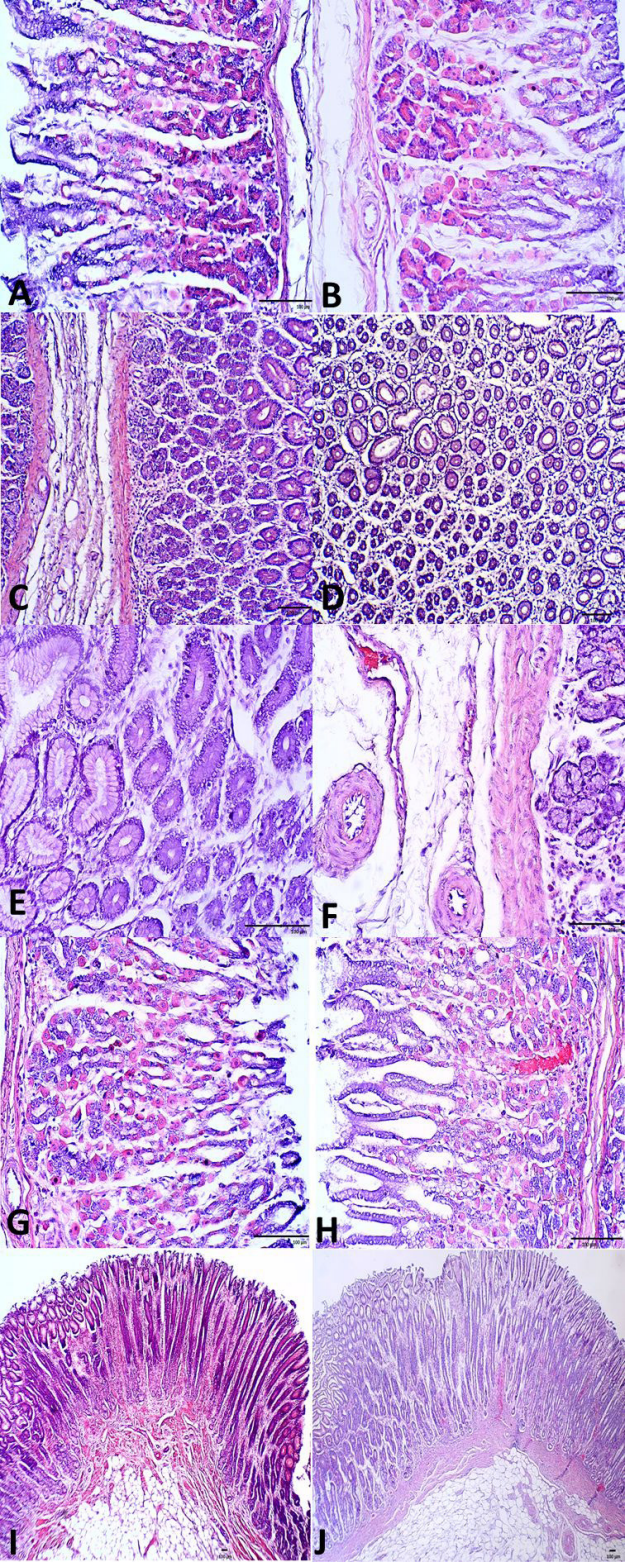
Optical microscopy photograph comparing the different regions of the abomasum between the group of Holstein calves fed 8 l of milk replacer per day and *ad libitum* forage or concentrate. (A) Fundus — concentrate group. 20×; (B) Fundus — forage group. 20×; (C) Body × concentrate group. 10×; (D) Body — forage group. 10×; (E) Pyloric region × concentrated group. 20×; (F) Pyloric region — forage group. 20×; (G) Abomasal folds — concentrated group. 20×; (H) Abomasal folds — forage group. 20×; (I) Pyloric torus — forage group. 20×; (J) Pyloric torus — forage group. 20×.

**Figure 7:**
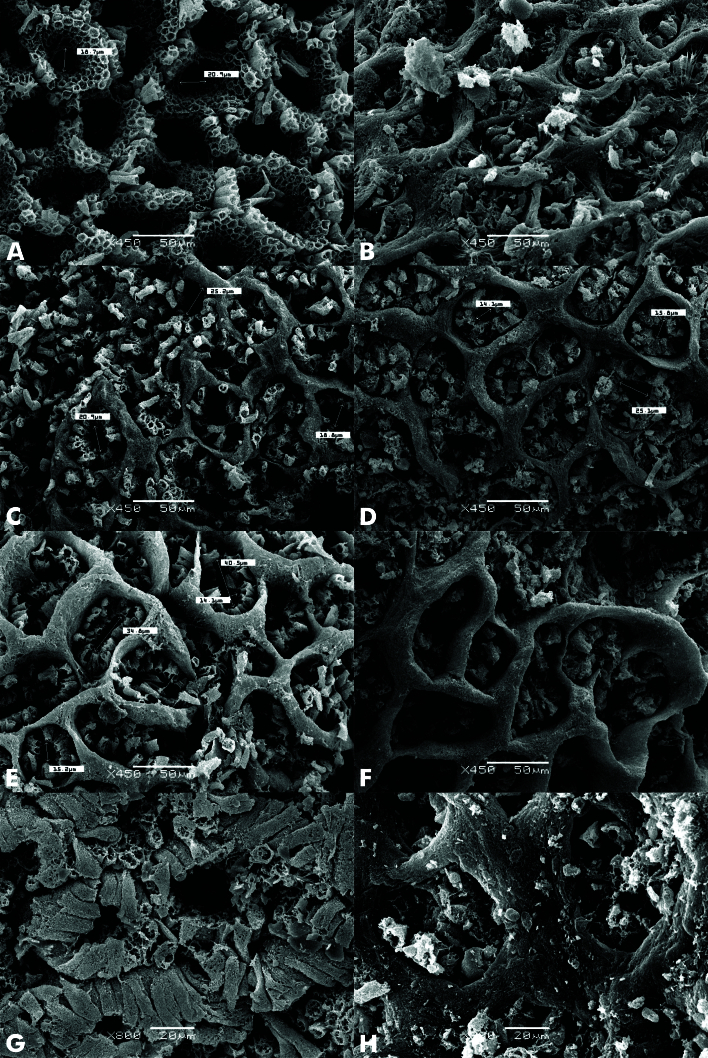
Electronic microscopy photograph comparing the different regions of the abomasum between the group of Holstein calves fed 8 l of milk replacer per day and forage or concentrate *ad libitum*. (A) Fundus — concentrate group. 450×; (B) Fundus — forage group. 450×; (C) Body — concentrate group. 10×; (D) Body — fodder group. 450×; (E) Pyloric region — concentrated group. 450×; (F) Pyloric region — forage group. 450×; (G) Abomasal folds — concentrated group. 800×; (H) Abomasal folds — forage group. 800×.

**Figure 8:**
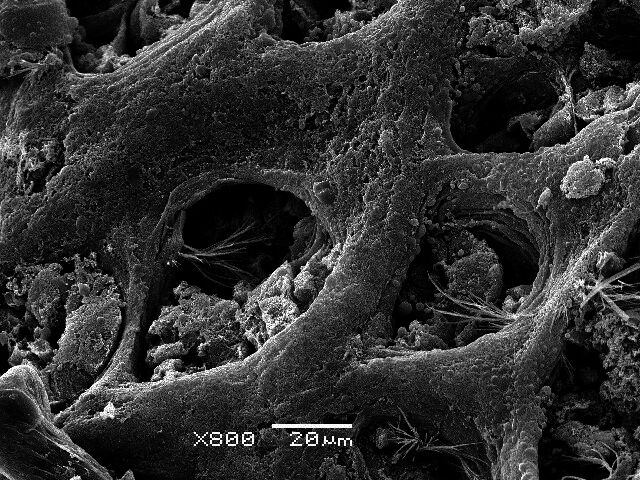
Electronic microscopy photograph of the abomasum of a Holstein calves fed 8 l of milk replacer per day and forage showing forage residues. 800×.

Histological analysis revealed four typical layers: mucosa, submucosa, muscle, and serosa (Figure 6). In all samples, the presence of glands was observed in both microscopic studies (optical and electron). The presence of glands, their pits, structures, and light are shown in Figures 6 and 7. The remaining hay was observed on the surface of the mucosa of the animals fed this diet (Figure 8). These glands were observed in the abomasal folds of both groups (Figures 6 and 7).

## Discussion

Comparing the morphohistological development of the abomasum of Holstein calves fed alfalfa hay or concentrate *ad libitum* with 8 l of milk substitute per day revealed no significant differences between the two groups. This may be because although the calves supplemented with hay had a lower intake for both diets, MR was the main feedstuff ingested, contributing to a well-balanced nutrition (Amaro et al., 2024).

Chamois (*Rupicapra pyrenaica*), ibex (*Capra pyrenaica*), and sheep (*Ovis aries*) fed different types of hay showed significant differences in the values of the weight of the empty abomasum in absolute terms. Function of the empty live weight and the weight of the complete digestive system, as well as the content of the abomasum in grams, were significantly higher in sheep than in chamois, with the ibex occupying an intermediate position (Lavín & Mantecón, 1993). However, in our study, no significant differences in the weights of the full and empty abomasum were observed between the hay-fed group and concentrate-fed group. This could be attributable to the diets being similar in percentage of CP and the consumption of dairy supplements remaining constant and low in both groups of calves.

Sarma and Bhattacharya (1997) have reported that the average total number of abomasal folds was 16.67 in zebu, 15.00 in mithun, and 14.33 in yak, being relatively more in zebu indicating increased absorptive area in this type of animals. Among the calves studied, the average number of folds was 15.3 in the hay-fed group and 16.4 in the concentrate-fed group. That is according to the description that cattle have more than 12 abomasal folds (Dyce et al., 2009).

The pyloric torus is a fibromuscular protuberance with numerous fat cells (Lauwers et al., 1979). The absence of pyloric torus development observed in three calves (one from the hay-fed group and two from the concentrate-fed group) may be attributed to individual variability, and no future implications for digestive tract functionality were anticipated.

Three glandular regions have been described at the bovine abomasum: cardiac, fundic, and pyloric. Secretory cells were observed in all regions of the abomasum. The thickness of the layers of this organ increases in the pyloric region (Aage et al., 2007). In this study, we described glands in all parts of the abomasum, but the development and quantity varied between regions and diets; differences were observed in the presence of gastric pits (their presence was greater in calves fed with concentrate), which could be explained by their ease of digestion compared to hay. Scanning electron microscopy studies have been conducted on sheep to evaluate alterations caused by infestation with *Haemonchus* (Nicholls et al., 1985), camelids (*Camelus dromedarius*) to describe their normal structure (Raji, 2011), sheep (*O. aries*) (Movahedizadeh et al., 2018) and goats (*Capra hircus*) (Mahesh et al., 2017). However, to the best of our knowledge, no previous study has evaluated the abomasum of calves.

In studies performed in goats and lambs fed diets with increasing tannin concentrations, a positive correlation was observed between the increase in the amount of tannins, thickness of the abomasal epithelium, and degree of keratinization of the abomasum. In addition, the number of parietal cells decreased as the tannin content increased. This may affect the secretory function of the glands and prevent proper protein digestion in this organ (Kia et al., 2016; Mbatha et al., 2002). The tannin concentrations in the feeds used in this study were insufficient to assert that the observed differences were caused by the varying tannin concentrations between the diets, as both diets were associated with very low tannin contents.

This is the first report on the anatomical characterization of abomasal morphology in young dairy calves. No significant differences were observed in the data obtained when comparing both groups of calves fed 8 l of MR with free access to concentrate feed and those fed high-quality alfalfa hay, likely because of the balanced nutritional contribution of both diets. However, microscopic examination suggests that differences in abomasal morphology may exist at the microscopic level. Further studies are required to confirm these differences and determine any significant future implications.
